# Osteocalcin and serum insulin-like growth factor-1 as biochemical skeletal maturity indicators

**DOI:** 10.1186/s40510-017-0184-y

**Published:** 2017-10-02

**Authors:** Tulika Tripathi, Prateek Gupta, Priyank Rai, Jitender Sharma, Vinod Kumar Gupta, Navneet Singh

**Affiliations:** 1 Department of Orthodontics and Dentofacial Orthopaedics, Maulana Azad Institute of Dental Sciences, Bahadur Shah Zafar Marg, New Delhi, 110002 India; 2 Department of Biochemistry, Govind Ballabh Pant Institute of Postgraduate Medical Education and Research, Jawaharlal Nehru Marg, New Delhi, 110002 India

**Keywords:** Osteocalcin, IGF-1, Biochemical markers

## Abstract

**Background:**

With change in concepts of growth determination methods, there is a surge in the measurement of biomarkers for appraisal of growth status. Osteocalcin is a bone-specific protein and was observed to parallel the normal growth curve. Hence, the present study was intended to assess the levels of serum osteocalcin and serum insulin-like growth factor-1 (IGF-1) and compare them with cervical vertebral maturation index (CVMI) stages.

**Methods:**

The cross-sectional study was performed on 150 subjects (75 males and 75 females) in the age group of 8–20 years and segregated into six CVMI stages. Serum osteocalcin and IGF-1 were estimated by ELISA. Mann-Whitney *U* test was used to compare the mean ranks of serum osteocalcin and serum IGF-1 with different CVMI stages. Spearman correlation was performed to find association between serum osteocalcin and serum IGF-1 across six CVMI stages.

**Results:**

Peak serum IGF-1 levels were obtained at CVMI stages 4 and 3 for males and females, respectively, with insignificant difference between stages 3 and 4 in females. Peak serum osteocalcin levels were found at stage 5 and 3 for males and females with insignificant difference from other stages except stages 5 and 6 in males. A statistically significant correlation was seen between serum IGF-1 and serum osteocalcin across six CVMI stages (*P* < 0.01).

**Conclusions:**

Osteocalcin followed IGF-1 across all CVMI stages but showed insignificant interstage differences.

## Background

Successful application of various orthodontic treatment modalities depends on the skeletal growth status of the patient. Skeletal growth can be predicted via hand wrist and cervical vertebral maturation index (CVMI) stages, but it comes at an expense of radiation exposure to the patient and inherent limitation of variability in subjective assessment of the radiographs [1].

According to functional matrix theory, growth and development of skeletal units is governed by function of surrounding soft tissue matrix [2]. Craniocervical relationships are affected by the associated functions like respiration, digestion, speech, and equilibrium [3]. Further, it has been established that cervical column differs in various skeletal jaw relationships and pressure; morphology of facial components and body posture alter the height of vertebral bodies and hence, affect the reliability of CVMI [4–7].

With the changing concepts in growth determination methods, growth can be assessed via measurement of extracellular bone matrix constituents, which are molecular signatures of the skeletal growth process [8]. Insulin-like growth factor-1 (IGF-1) is one such matrix constituent, which has been profoundly researched for its role in both prenatal and postnatal skeletal growth. It facilitates growth under the action of growth hormone and is also directly stimulated by androgens to accelerate growth velocity [9]. IGF-1 receptor has even been localized to mandibular condylar cartilage, thus acting as a suitable candidate to evaluate the pubertal growth in the craniofacial region [10]. To consolidate the outcomes of research on IGF-1, we need to explore new bone-related factors, which may provide an insight towards precise skeletal growth assessment during puberty. Among all bone matrix constituents, only osteocalcin is unique to bone and can serve as a sensitive and specific serum marker for bone formation [11].

Further, bone formation depends on osteoprogenitor cell replication and differentiation into osteoblasts. Serum IGF-1 can simultaneously stimulate these two functions [12] and has been observed to stimulate osteocalcin synthesis in bone [13]. IGF-1 affects longitudinal bone growth while osteocalcin reflects activity of the whole skeleton and not just the sites of longitudinal growth [14–16]. Moreover, osteocalcin is a marker of late osteoblast differentiation [17] and was found to parallel the growth velocity curve [18], thus depicting a direct representation of process of bone formation.

Thus, the aim of the present study was to assess the levels of serum osteocalcin and serum IGF-1 and to compare it with CVMI stages. Furthermore, we intended to determine the significance of the marker levels between the two genders in different CVMI stages.

## Methods

This cross-sectional study was conducted on 150 subjects (75 males and 75 females) in the age group of 8–20 years who visited the Department of Orthodontics and Dentofacial Orthopaedics, Maulana Azad Institute of Dental Sciences, New Delhi, India. Subjects were enrolled in the study if they were in good general health. Subjects with growth abnormality, systemic disease, history of long-term medication, trauma, or surgical intervention in the area of cervical vertebra were excluded from the study.

Our study was approved by the research ethical committee of Maulana Azad Institute of Dental Sciences, New Delhi, India. Written informed consent was obtained from the subjects and their parents following explanation of the test procedure orally and through a bilingual patient information sheet.

The sample size appraisal was done at 5% significance level (*α* = 0.05). A minimum of ten subjects were required in each CVMI stage based on power of 80%. Personal information, history, and standardized lateral cephalograms were obtained for all subjects. Two examiners (TT and PG) independently evaluated the cervical vertebrae radiographic morphology on lateral cephalograms using the criteria of Hassel and Farman [19] and grouped the 150 selected subjects into six CVMI stages. A week later, similar radiographic evaluation was done by the same examiners for intra-examiner reliability.

Five milliliters of blood was collected from median cubital vein by venipuncture in red plain blood collection vials for each subject between 0900 and 1000 hours to avoid diurnal variations, and the timings were matched with taking of lateral cephalograms. Assessment of serum alkaline phosphatase, ionized calcium, and phosphorous was carried out for all the chosen subjects to further exclude any skeletal metabolic imbalance. Each blood sample was centrifuged at 4000 rpm for 15 min to separate the serum, which was then segregated equally by pipetting in two different plastic Eppendorf tubes (Eppendorf, Hamburg, Germany) and stored in different plastic boxes at −80°C for separate evaluation of IGF-1 and osteocalcin. Samples were prevented from repeated freeze/thaw cycles.

Serum IGF-1 ELISA (DRG International, USA) and Microvue Osteocalcin EIA (Quidel Corporation, CA, USA) kits were used to measure serum IGF-1 and osteocalcin levels, respectively. Serum samples were subjected to enzyme-linked immunosorbent assay based on competitive binding with anti IGF-1 and osteocalcin antibodies pre-coated on a 96-well plate. Using the mean absorbance values for each sample, corresponding concentrations were determined after plotting the standard calibration curves. The minimum analytical detection limits for serum IGF-1 and osteocalcin assays were 9.75 and 0.45 ng/ml, respectively.

Statistical analysis was carried out with SPSS software for Windows (version 23.0; SPSS, Chicago, Ill). The Kruskal-Wallis and Mann-Whitney *U* tests were used to compare mean ranks of serum IGF-1 and osteocalcin among different CVMI stages. Comparison between males and females for each CVMI stage was also performed with the Mann-Whitney *U* test. The Spearman correlation test was performed to find relationship between serum IGF-1 and osteocalcin values at different CVMI stages. Kappa statistic was used to measure inter-examiner and intra-examiner reliabilities in staging of lateral cephalograms.

## Results

Kappa statistics showed no significant difference in inter-examiner and intra-examiner readings (0.92 and 0.94, respectively).

**Fig. 1:**
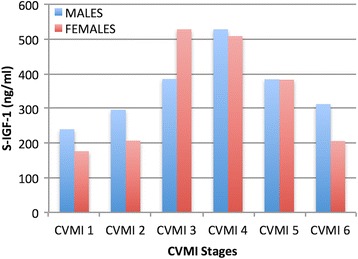
The distribution and comparison of mean serum IGF-1 levels in males and females in the six CVMI stages

Mean serum IGF-1 levels in males increased progressively from lowest value (239.79 ng/ml) at CVMI stage 1 to peak value at stage 4 (528.03 ng/ml) followed by decline towards stage 6. It was observed that CVMI stages 3 and 5 had comparable means. In females, IGF-1 levels rose from minimum levels (175.81 ng/ml) at stage 1 to peak (527.50 ng/ml) at stage 3. The levels declined from stage 3 to stage 6 (Table 1). Peak values for both females and males were comparably similar while minimum values were smaller for females as compared to males (Fig. 1).

**Table 1:** Serum IGF-1 (ng/ml) descriptive statistics for each CVMI stage in males and females

	Males					Females						
CVMI	*N*	Age	Range	Mean ± SD	Median	N	Age	Range		Mean ± SD		Median
1	12	10.25	122.50	400.00	239.79 ± 94.48	238.75	12	8.75	157.90	189.20	175.81 ± 10.46	176.75
2	12	11.50	130.00	457.50	295.79 ± 97.92	325.50	12	10.92	193.80	224.20	207.38 ± 9.75	207.37
3	13	13.46	215.00	600.00	384.61 ± 139.84	325.00	14	13.29	375.00	600.00	527.50 ± 87.93	562.50
4	14	14.71	257.50	600.00	528.03 ± 114.15	600.00	13	14.46	157.50	600.00	507.88 ± 153.10	600.00
5	12	15.42	250.00	600.00	384.16 ± 13.213	325.00	12	15.33	300.00	530.00	382.75 ± 70.94	372.50
6	12	17.75	93.80	400.00	312.60 ± 97.03	325.00	12	18.92	197.60	220.90	205.81 ± 6.64	206.44

**Fig. 2:**
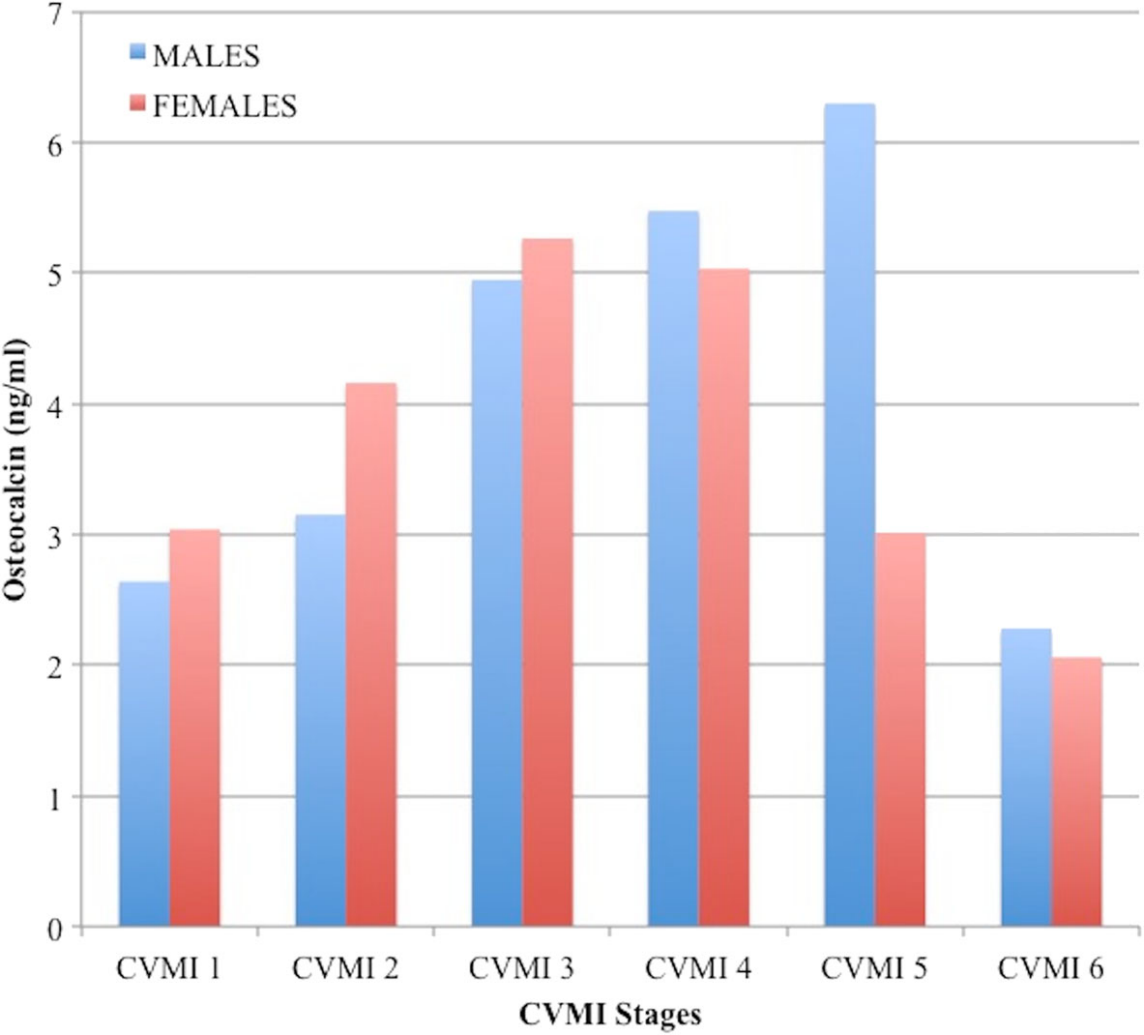
The distribution and comparison of mean serum osteocalcin levels in males and females in the six CVMI stages

Table 2 summarizes the mean values and ranges of the osteocalcin in males and females, respectively. In males, osteocalcin showed gradual increase from stage 1 to highest level (6.29 ng/ml) at stage 5 followed by sudden decline to reach lowest value (2.28 ng/ml) at stage 6. In females, osteocalcin gradually increased from stage 1 to reach highest levels at stage 3 (5.26 ng/ml) and declined thereafter. The peak value for males was found to be higher than that of the peak value for females (Fig. 2).

**Table 2:** Osteocalcin (ng/ml) descriptive statistics for each CVMI stage in males and females

	Males					Females						
CVMI	*N*	Age	Range	Mean ± SD	Median	*N*	Age	Range		Mean ± SD		Median
1	12	10.25	1.12	4.82	2.64 ± 1.03	2.47	12	8.75	0.90	5.39	3.04 ± 1.32	2.99
2	12	11.50	1.56	5.41	3.15 ± 1.16	3.02	12	10.92	1.33	7.80	4.16 ± 1.93	3.82
3	13	13.46	1.65	16.10	4.94 ± 4.21	2.60	14	13.29	1.85	20.20	5.26 ± 4.90	3.75
4	14	14.71	1.80	10.40	5.47 ± 2.91	5.60	13	14.46	1.80	10.00	5.03 ± 2.65	4.40
5	12	15.42	2.15	11.90	6.29 ± 3.71	5.80	12	15.33	1.90	7.40	3.01 ± 1.60	2.35
6	12	17.75	1.06	4.46	2.28 ± 1.08	1.98	12	18.92	0.80	3.54	2.06± 0.87	1.92

The Kruskal-Wallis test showed statistically significant differences in mean ranks of serum IGF-1 and osteocalcin levels among CVMI stages. It denoted variable distributions across the six CVMI stages.

The Mann-Whitney *U* test performed to reveal interstage differences in serum IGF-1 values showed that in males, CVMI stage 1 had statistically significantly lower value than at stage 3 (*P* < 0.01), stage 4 (*P* < 0.001), and stage 5 (*P* < 0.01) while CVMI stage 4 IGF-1 value was significantly higher than all other CVMI stages (*P* < 0.05). No significant differences were observed among CVMI stages 1, 2, and 6 (Table 3).

**Table 3:** Kruskal-Wallis and Mann-Whitney *U* tests comparing each CVMI stage in males and females for serum IGF-1 and serum Osteocalcin

		Serum IGF-1				Osteocalcin			
		Kruskal-Wallis test on serum IGF-1 vs CVMI is significant: *P* < 0.0001				Kruskal-Wallis test on osteocalcin vs CVMI is significant: *P* < 0.0001			
		Mann-Whitney *U* tests comparing the mean ranks of serum IGF-1 for CVMI stage a vs b				Mann-Whitney *U* tests comparing the mean ranks of osteocalcin for CVMI stage a vs b			
CVMI stage a	CVMI stage b	Difference in location (mean): a-b		*P* value		Difference in location (mean): a-b		*P* value	
		Males	Females	Males	Females	Males	Females	Males	Females
1	2	−56.00	−31.57	0.114 (NS)	0.0001	−0.51	−1.12	0.273 (NS)	0.175 (NS)
	3	−144.82	−351.68	0.011	0.0001	−2.30	−2.22	0.277 (NS)	0.237 (NS)
	4	−288.24	−332.07	0.001	0.0001	−2.83	−1.99	0.007	0.064 (NS)
	5	−144.37	−206.93	0.014	0.0001	−3.65	0.03	0.013	0.707 (NS)
	6	−72.81	−29.99	0.114 (NS)	0.0001	0.36	0.98	0.273 (NS)	0.043
2	3	−88.82	−320.11	0.225 (NS)	0.0001	−1.79	−1.10	0.663 (NS)	0.959 (NS)
	4	−232.24	−300.50	0.0001	0.0001	−2.32	−0.87	0.031	0.463 (NS)
	5	−88.37	−175.36	0.291 (NS)	0.0001	−3.14	1.15	0.043	0.094 (NS)
	6	−16.81	1.57	0.514 (NS)	0.713(NS)	0.87	2.10	0.065 (NS)	0.004
3	4	−143.42	19.61	0.008	0.867(NS)	−0.53	0.23	0.382 (NS)	0.662 (NS)
	5	0.44	144.75	0.852 (NS)	0.0001	−1.35	2.25	0.231 (NS)	0.157 (NS)
	6	72.01	321.68	0.347 (NS)	0.0001	2.66	3.20	0.044	0.006
4	5	143.86	125.13	0.011	0.019	−0.82	2.02	0.554 (NS)	0.053 (NS)
	6	215.43	302.07	0.0001	0.0001	3.19	2.97	0.002	0.001
5	6	71.56	176.93	0.319 (NS)	0.0001	4.01	0.95	0.002	0.053 (NS)

In females, CVMI stage 1 IGF-1 value was statistically significantly lower than all other CVMI stages (*P* < 0.0001). CVMI stage 2 value was significantly lower than CVMI stages 3, 4, and 5 (*P* < 0.0001) but significant difference was not observed with CVMI stage 6. CVMI stage 3 value was significantly higher than all CVMI stages (*P* < 0.0001) except stage 4 (Table 3).

Inter-CVMI stage difference by performing the Mann-Whitney *U* test for osteocalcin showed that osteocalcin values increased gradually till stage 5 with insignificant difference between adjacent stages and declined statistically significantly at stage 6 (*P* < 0.01) in males whereas in females, though it was a progressive rise till stage 3 followed by steady decline till stage 6, difference was insignificant between adjacent stages (Table 3).

Significant difference between the IGF-1 levels in males and females was observed at CVMI stages 2 (*P* < 0.01), 3 (*P* < 0.01) and 6 (*P* < 0.0001) while the difference was not significant in other stages (Table 4). Results of the Mann-Whitney *U* test between males and females revealed a statistically significant difference in osteocalcin levels at CVMI stage 5 (*P* < 0.05) (Table 4).

**Table 4:** Mann-Whitney test for the comparison of serum IGF-1 and osteocalcin between different CVMI stages of males and females along with the *P* value

			Serum IGF-1			Osteocalcin		
			Male	Female	*P* value	Male	Female	*P* value
CVMI	1	N	12	12	0.22 (NS)	12	12	0.488 (NS)
		Mean ± SD	239.79 ± 94.48	175.81 ± 10.46		2.64 ± 1.03	3.04 ± 1.32	
		Median	238.75	176.75		2.47	2.99	
	2	N	12	12	0.01	12	12	0.149 (NS)
		Mean ± SD	295.79 ± 97.92	207.38 ± 9.76		3.15 ± 1.16	4.16 ± 1.93	
		Median	325.50	207.38		3.02	3.82	
	3	N	13	14	0.007	13	14	0.680 (NS)
		Mean ± SD	384.62 ± 139.84	527.50 ± 87.94		4.94 ± 4.21	5.26 ± 4.90	
		Median	325.00	562.50		2.60	3.75	
	4	N	14	13	0.90 (NS)	14	13	0.716 (NS)
		Mean ± SD	528.04 ± 114.15	507.88 ± 153.10		5.47 ± 2.91	5.03 ± 2.65	
		Median	600.00	600.00		5.60	4.40	
	5	N	12	12	0.55 (NS)	12	12	0.024
		Mean ± SD	384.17 ± 132.13	382.75 ± 70.94		6.29 ± 3.71	3.01 ± 1.60	
		Median	325.00	372.50		5.80	2.35	
	6	N	12	12	0.00	12	12	0.686 (NS)
		Mean ± SD	312.60 ± 97.03	205.81 ± 6.65	01	2.28 ± 1.08	2.06± 0.87	
		Median	325.00	206.44		1.98	1.92	

Spearman correlation coefficient (*ρ*) was used to determine the correlation between IGF-1 and osteocalcin across CVMI stages. A positive correlation was observed between IGF-1 and osteocalcin during the entire CVMI staging and was noted to be significant for both males and females combined at CVMI stages 1 to 6 (*P* < 0.001), 3 to 6 (*P* < 0.001), 4 to 6 (*P* < 0.001), 1 to 5 (*P* < 0.01), and 5 to 6 (*P* < 0.01) (Table 5). Further, correlation was statistically significant between IGF-1 and osteocalcin for all ranges of CVMI stages (*P* < 0.05) in males and for CVMI stages 3 to 6 (*P* < 0.05) and 4 to 6 (*P* < 0.05) in females (Table 5).

**Table 5:** Spearman coefficient of correlation between serum IGF-1 and osteocalcin for different CVMI stages

	Serum IGF-1 vs osteocalcin								
	Overall			Male			Female		
CVMI stage	Spearman correlation	*P* value	*n*	Spearman correlation	*P* value	*n*	Spearman correlation	*P* value	*n*
1 to 6	0.302	0.000	150	0.459	0.000	75	0.155	0.185 (NS)	75
1 to 3	0.225	0.053 (NS)	75	0.344	0.037	37	0.181	0.277 (NS)	38
3 to 6	0.423	0.000	102	0.527	0.000	51	0.308	0.028	51
4 to 6	0.410	0.000	75	0.420	0.009	38	0.334	0.043	37
1 to 5	0.252	0.004	126	0.464	0.000	63	0.067	0.602 (NS)	63
5 to 6	0.424	0.003	48	0.405	0.049	24	0.339	0.105 (NS)	24

## Discussion

Growth assessment of the craniofacial region is a subject of interest for the clinicians, researchers, and scientists. Observational studies involving analysis of series of cephalometric films and the behaviour of cells and extracellular matrix molecules are the methods employed in the past two decades to gain information about growth and growth factors [8]. Radiographic methods though popular, have limitations in clinical applicability. Thus, the task of providing information about growth is entrusted to biomarkers whose levels correlate among different body fluids at particular growth stage as depicted by Sinha et al. for IGF-1 [20].

Until now, IGF-1 has proved to be comparably authentic in representing growth status as compared to other biomarkers but disagreement still remains, which initiated the need for exploring the potentiality of other biomarkers. Osteocalcin was employed in our study as it is the most abundant non-collagenous protein of the bone matrix and is exclusively associated with bone [11].

In the 1990s, osteocalcin was established as a marker of bone formation and bone turnover [21]. Subsequently, with the discovery of osteocalcin’s expression in differentiated osteoblasts, its role in growth has continuously been elucidated.

Osteocalcin, a vitamin K-dependent protein undergoes carboxylation to bind hydroxyapatite in bone and has higher affinity for calcium, thus facilitating bone mineralization [22]. Thus, circulating osteocalcin was assayed using two-site ELISA in our study to assess the growth status during puberty.

Results in our study depicted that IGF-1 and osteocalcin showed similar changes with skeletal maturation as pubertal growth curve. Peak values for IGF-1 were observed at CVMI stage 4 in males and CVMI stage 3 in females. Many previous studies observed similar results for both males [23, 24] and females [23, 25–27] except Masoud et al. [28] who discovered peak values at CVMI stage 5.

Peak osteocalcin values were found at CVMI stage 5 in males and CVMI stage 3 in females. Studies on mice have provided evidence of the role of osteocalcin in increasing testosterone production in males by feedforward loop [29]. Moreover, higher serum testosterone levels found in late puberty [30] and significant positive correlation between osteocalcin and testosterone as reported by Johansen et al. [18] might be the cause of peak osteocalcin levels observed at CVMI stage 5 in males in our study. Earlier peak in females might be linked to rising oestrogen levels in late puberty as oestrogen causes growth plate closure and inhibit periosteal apposition [30, 31]. Also, osteocalcin levels peaked 2 years earlier in females (13.29 years) as compared to males (15.33 years), which corresponded to the previous studies [29, 32–34].

During puberty, bone grows both longitudinally and in cortical thickness. Whereas longitudinal bone growth was reported to cease after mid puberty, cortical thickening continues in late puberty [30]. Further, osteocalcin has been observed to be high in cortical bone in comparison to trabecular bone [35, 36]. Such site specificity with significant influence of oestrogen is pivotal for observed sexual dimorphism in skeletal growth. Moreover, higher peak osteocalcin values observed in males as compared to females might be associated with greater cortical thickness observed in males as compared to females [37]. Thus, different mechanisms with complex interactions between associated biomarkers may regulate bone growth during puberty.

It was reported by Johansen et al. [18] that osteocalcin correlated significantly with IGF-1. In our study, a positive correlation was observed between osteocalcin and IGF-1 across CVMI stages, which was highly significant in all the stages in males but from CVMI stage 3 to stage 6 in females. Thus, in males, osteocalcin and IGF-1 followed the same trend across all CVMI stages whereas in females, a similar trend was observed only after peak levels. Such gender-based correlation involving particular pubertal stages was not emphasized in previous study [18]. Moreover, the increase in osteocalcin levels on IGF-1 administration indicates a role of IGF-1 on osteocalcin regulation as reported by Johansson et al. [38].

Inter-CVMI stage differences revealed that osteocalcin levels gradually rise in both males and females while suddenly decline in males from CVMI stage 5 to CVMI stage 6 which is in contrast to more gradual decline in females from CVMI stage 3 to CVMI stage 6. Such gender variation in osteocalcin during puberty suggests some sex hormone regulation of osteocalcin levels. On the contrary, IGF-1 showed significant difference in peak levels at CVMI stages 3 and 4 from other CVMI stages.

Gender differences for osteocalcin at each CVMI stage were not found to be significant as there was a gradual rise in both males and females except stage 5 when males attained peak values and females showed declining levels. Nevertheless, both males and females exhibited lowest osteocalcin values at the completion of growth in CVMI stage 6. Conversely, IGF-1 showed significant difference between males and females at CVMI stages 3 and 6 with stage 3 values higher in females and stage 6 values higher in males. It demonstrated attainment of earlier peak in females and prolonged growth spurt in males. Thus, our results signify that IGF-1 was still higher in males as compared to females at CVMI stage 6 when osteocalcin levels were lowest and comparable in both males and females. This might have resulted due to IGF-1 regulation of both bone and muscle growth [39]. According to Xu et al. [39], muscle growth peak later than bone growth, which might be the reason for higher IGF-1 values even in CVMI stage 6 as compared to CVMI stage 1. While at the same time, osteocalcin values declined to its lowest owing to its bone specificity. Gender differences in IGF-1 values observed in late CVMI stage could be explained from findings of Neu et al. [40] who described gender-specific course in muscle growth during puberty with males demonstrating prolonged growth as compared to females.

Further, when IGF-1 and osteocalcin were compared, peak values were observed at different CVMI stages in males, which pointed towards the role of some other male specific factor, which is regulating osteocalcin levels during puberty and may be attributed to the longer period of cortical growth [30].

Growth hormone is the common hormone, which stimulates skeletal growth both via facilitating IGF-1 release and might be involved in production of osteocalcin in osteoblasts [41–44]. In the present study, both the markers indicated skeletal maturity. Although IGF-1 receptors have been identified in mandibular condyle and local production of IGF-1 proceeds in parallel to availability to IGF-1 receptors, role of osteocalcin status in growth regulation of mandibular condyle is still to be authenticated with evidence [45, 46]. Orthodontics relies significantly on the status of skeletal growth, which must be substantiated with backing in the form of multiple biomarkers. Further research is required to ascertain whether a particular marker is better than any other marker to depict growth status or different markers yield distinct data at a particular growth stage.

## Conclusions

1. Serum IGF-1 showed peak levels at CVMI stage 4 and stage 3 in males and females, respectively, with statistically significant interstage differences except stages 3 and 4 in females.
2. Serum osteocalcin peak values were observed at CVMI stage 5 and stage 3 in males and females, respectively, but with statistically insignificant interstage differences except stages 5 and 6 in females.
3. A statistically significant correlation was obtained between serum IGF-1 and osteocalcin across all six CVMI stages.
4. A statistically significant difference in serum IGF-1 values was observed between males and females at CVMI stages 2, 3, and 6 whereas for osteocalcin at CVMI stage 5.

Contribution of IGF-1 in growth process is important and could serve as a practically possible marker to assess growth status. In the light of present findings, role of osteocalcin in skeletal growth cannot be neglected considering its bone specificity and its accompanying of growth curve. Future research involving hormones and bone-related factors with greater sample size in which subjects should be longitudinally assessed would uncover the complexity of pubertal growth regulation and increase our understanding of facts still inexplicable at present.
